# Outlines of the Institutes of Medicine; Founded on the Philosophy of the Human Economy, in Health and Disease

**Published:** 1841-02

**Authors:** 


### REVIEWS.

---

Art. VII.—*Outlines of the Institutes of Medicine; founded on the Philosophy of the Human Economy, in health and disease. In three parts.*

"Should we build facts upon facts, until our pile reached the heavens, they would tumble in pieces, unless they were cemented by principles.—Rush."

By Joseph A. Gallup, M. D.—*Author of sketches of epidemic Diseases in the State of Vermont*; late Professor of Theory and Practice in the Vermont Academy of Medicine, and of the Clinical school of Medicine; Ex-President of the Vermont Medical Society; Hon. Member of the Medical Society of the State of New York, &c. Boston: Otis, Boarders & Co. 1839.

Should any of our readers allege to us that this title-page presents an aspect somewhat out-of-the-way and uncommon, we should hardly be so uncivil as to contradict them. Instead of thus unceremoniously plunging into collision with them at first sight, in either thought or feeling, we should be more likely perhaps to reply, "*so much the better.*" We like consistency and aptitude; and on that principle, this uncommon title-page very fitly ushers to the world a brace of "uncommon" volumes. For such is the character of the work before us.

We have not however yet said, nor do we indeed design to say, whether it is uncommonly *good*, or uncommonly *bad*—or whether it is either the one or the other. We insist only that it is uncommon. On the score of its quality, our readers may, at pleasure, decide for themselves, by aid of the means to that effect, with which we shall furnish them; or, better still, by *purchasing* the books, as we have done, and reading them *verbatim*, from first to last.

The production before us, we repeat the third or fourth time, is uncommon; and the following are some of the particulars which make it so. It manifests in its author an uncommon amount and accuracy of reading, remembering, and recording, and uncommon earnestness in thinking, and in his propensity for theorizing. *His* thoughts and theories however are not always *ours*. But that matters not; they may be better than ours; and we shall not therefore condemn either them, or him on account of them, nor even find fault with them. Let their trial be held at the tribunal of the public—and fairness and justice will there be awarded them.

The work is farther uncommon on account of its old-fashioned elaborateness and formality—the number and brevity of its "Sections" and subdivisions, furnishing the reader with all possible convenience on the score of stopping, breathing, and reflecting places,—its abundant and perhaps unnecessary employment of certain words already existing, but almost out of use, and of its ready coinage of new ones, (the author proclaiming the entire insufficiency of the present meagre nomenclature of medicine to answer the demand of *improvement* and *discovery*)—and its rich store of facts, many of them sufficiently interesting and useful, and of opinions and notions not quite so valuable. To these may be added an uncommon proneness in the Doctor to the labour of "*definition*", in which complete success is never attained by mortals, (the faculty and language of definition, according to an ancient sage, "*belonging only to the gods*") and an effort at uncommon precision in all things. Nor shall we charge the effort with fault or failure, except in the article of style, which we cannot admit to be, in every instance, either precise or classical, perspicuous or chaste. Yet it is generally intelligible; and we have reason to believe that our author has laboured it as sedulously and intensely, and esteems it himself at least as highly, as any other attribute of his books.

Such are a few of the grounds (and many more might be adduced) on which we have pronounced the "Outlines" uncommon; but have not on that account either praised them or blamed them. And we shall only add, under the present head of our remarks, that although every uncommonness, in science or letters, does not necessarily imply improvement or discovery ;yet the reverse is not true. Every discovery and improvement must have something uncommon in it, else it would be in no shape an amendment.

But from the uncommon we must pass to the common and general qualities of the "Outlines", by which our readers will be much better enabled to judge of their merit. To speak more definitely on the subject; we shall now give a brief view of the plan which our author has pursued in writing them, followed by a few extracts from them, a careful perusal of which, next to a perusal of the entire production, will furnish our readers with the best means of working out a correct decision respecting their character.

Covering by it as he does, the entire ground of the Institutes or Philosophy of Medicine, our author has thrown his work into three grand "Divisions"; those of Physiology, Pathology, and Therapeutics; and in his treatment of each branch he has incorporated not only the result of his own observation, and reflection, but also much that he has derived from a long-continued and toilsome examination of the productions of many of the most distinguished medical writers of all countries, and of former times, as well as of the present. The amount of matter therefore, which he has amassed in his publication, into whatever shape his taste and fancy may have led him to mould it, or whatever covering of unusual and fresh-coined words he may have thrown around it, can hardly fail to be ample and useful. To convince the reader that Dr. Gallup knows how to begin his fabric at the beginning, and construct it from the foundation *upward*, as the case requires, instead of following the example of some who build from the roof downward, we submit to his inspection the following extract, under the several heads or subdivisions of "Ultimate Composition", "Organic Composition," and a few others.

*Ultimate Composition.*

"The process of destructive analysis of animal bodies exhibits various substances; such as phosphorus, the phosphate of lime, soda, magnesia, potash, manganese, sulphur, chlorine, iron, carbon, silex, alumine, and several neutral salts in small proportion. However, the phosphate of lime is very abundant, entering into the composition of the bones and giving them their solidity."

"By analysis of the body, either spontaneously or by art, are found the following gaseous substances, making parts of the constitueut elements; oxygen, hydrogen, and azote, which may be confined. There are also, certain evanescent essences, in the living state, not confinable; as caloric, light, electric fluids, various gases, and the principle of *vitality*. These have more or less feeble combination with living organic bodies, and become evanescent before decomposition takes place. The principal elementary ingredients, which compose all the soft part in animal substances, are carbon, oxygen, hydrogen, and azote, in different proportions in the different organized elements; phosphorus and lime are the basis of the hard parts. Azote exists in the largest proportion."

"Chemico-physiological researches indicate many other substances in the human body, which some think have been constituents; yet they rather ought to be esteemed products, or secondary formations of vital organic influences. Many acids are found, as the sulphuric, carbonic, benzoic, uric, amniotic, oxalic, formic, acetic, malic, lactic, and rosacic, which last is found in lateritious sediment of the urine in fevers."

### "*Organic Composition.**

"Upon a cursory inspection of the human body, it is found to give a moderate resistance to the touch; to be composed of what may be styled plates, or animal membranes of different degrees of compactness, and pierced in all directions by apertures of very different calibres; these constitute the *solids.* Within these openings or tubes, are discovered liquids, either red or white, which pass in different directions through the solid parts; these are called the *fluids.* It would appear at first view, that the solids exceed the fluids by weight; but, it is found on desiccating the body, that the fluids exceed the solids as about nine to one. However, they are quite different in different subjects.

"The diffuse, invisible, actuating principle of animal bodies has its residence in both these solid and fluid substances, but is most eminently appreciable in the former. However, the fluids sustain an important office in the economy of the system, and bear around in their circuits the rich fluid animal matter that becomes attached to the solids to increase their growth, or supply their waste.

It appears to be ascertained at the present time, that there are at least four primary animal formations, which by coalescing in various proportions, constitute the organization of animal bodies;—these are *gelatin*, *albumen*, *fibrin*, and *mucus*; and some old *ozmazome.* These being vitally anima-

*The author wishes here to advise the reader, that his course in this arduous undertaking is intended to be a straight forward one; he cannot stop, *on ordinary occasions*, to give credit to various writers who may have richly contributed to improve the science of Medicine; and indeed, he scarcely feels able, in numerous particulars, to designate, whether the deductions he may advance have been formed by himself, or derived from others. Neither can he often stop to refute what he may suppose to be erroneous conclusions of others, and thereby interlard the work with contraries; but will endeavor to be satisfied, if he can arrange, and express his views in a manner to be rightly understood throughout the work.

lized molecules themselves, unite by a sort of peculiar formative affinity in the production of lines, or fibres; and in their turn these lines unite, by various crossings and interlacings, in the formation of tissues, or membranes. Again, these last, by a sort of plastic, formative impetus, generate what are called organs. If the process is not so formed as first to form lines as suggested, they are found in such a state, although the parts may have been formed in tissues by a gradual accretion, or elongation. Each of these primary animal elements merits some farther consideration.

"*Gelatin.*

"All the four animal constituent molecules before mentioned, are composed of carbon, hydrogen, oxygen, and azote, but in different proportions. Some physiologists have said, that gelatin does not possess any azote in its composition. But it seems now proved that it does, but not to the amount contained in fibrin. It is contended by some also, that gelatin is not found in the circulating mass of blood; however, this is shown to be a mistake, but it is found only in a small proportion.* The muscles, it appears, are first formed in the embryo, of gelatin, and afterwards recieve an addition of azote, by which they are converted into fibrin, which renders them more capable of contractility.

"Gelatin, or jelly, is extracted by boiling, from the tendons, bones, capsules of the joints, dura mater, cellular tissue, and all those membranes called by M. Pinel the fibrous membranes, of all which it is their principal constituent. They are called *fibrous* on account of their filamentary appearances. All these tissues seem to be formed by a condensed and modified state of the laminated or cellular tissue, for this is almost wholly formed of gelatin.

"It is represented by many experimenters, who have minutely investigated the subject, that the molecules of gelatin are not so large as those of fibrin. The tissues of which it is a principal constituent, possess a contractile property, but not so much so as tissues formed of either albumen, or of fibrin. It is probable, however, further observation may be required in order to be satisfied, whether gelatin or albumen possesses the most contractility. That all the tissues are endowed with more or less contractility is very manifest, otherwise they could not aid in the organic functions.

---

*Berzelius.

"The physical proporties of gelatin are; 1st. It is soluble in water, but not in alcohol. 2d. It liquifies by heat, whilst albumen hardens by heat. 3d. It can be made solid by cold, and drying, but readily putrifies with water. 4th. It may be again liquified by heat, whilst albumen cannot be restored when once coagulated. 5th. It has a chemical affinity with tannin, and unites with it, by which union it becomes unliquifiable and insoluble. 6th. Uniting with tannin from the bark of astringent vegetables, in the skin of animals, it converts them into a dense, elastic state called leather.

"*Albumen.*

"This constituent of animal matter, receives it name from the albumen of the white of eggs, to which it is very similar. M. Cloquet says, it always contains sulphur, and subcarbonate of soda, besides the common elementary constituents. When in a fluid state it coagulates at about 165° of Fah. Alcohol, the acids, muriate of mercury, astringents, &c., coagulate it. Subjected to the process of drying, after being coagulated, it forms a solid semi-transparent substance resembling horn, insoluble except sparingly by alkalies.

"This substance is very abundant in nature, as it enters into the composition of both animal and vegetable bodies. In the human system it is found abounding in the serum of the blood, in the humours of the eye, and most of the secretions, particularly in the milk; and, also, in the white tissues. It is esteemed to be a principal constituent of the nervous pulp, throughout the brain and nerves; also of the glandular textures. It is easily extracted from other substances in combination, and its most delicate test is a solution of muriate of mercury.

"It exists abundantly in the seeds of vegetables, as well as in their roots, stalks, and leaves, It is, likewise, an important constituent of most of the tribes of insects, reptiles, and crustaceous animals, as well as the infusoria. As it is readily decomposed when subjected to warmth and moisture, it affords various volatile substances in the form of effluvia, more or less disagreeable, if not obnoxious. Old animals have in their composition the most albumen, whilst young ones possess the most gelatin. Seguin has shown it to be the most essential principle in the production of spontaneous fermentation of vegetable substances, thereby evolving saccharine, alcoholic, and acetic products. This substance is of much use in clarifying liquids; and, also, by inviscating poisonous substances in the stomach, may disarm them of their deleterious influences."

The following is our author's somewhat curious, though not *original* speculation, respecting the *primitive formation* of living organized beings. Nor is it designed by him to contravene or question the Mosaic account of the process. It is only intended as a comment on that account, referring to it as authority, and offering an exposition of it.

"This power, this property, may hence continue to be known by the appellations of vital principle, vital force, vital energy, or simple animation, or life. Its peculiar influences in the human system may hereafter receive much amplification.

"*Its Origin and Source.*

If it should be inquired, from whence originated this principle;—it might be answered by retorting this inquiry, whence originated light, affinity, gravity, and other properties and essences in nature? That creative Power who said, "Let there be light and there was light," said also, "Let us make man in our own image,"—"and breathed into his nostrils the breath of life, and man became a living soul."* If the *breath* used in the narration should be considered a figurative expression, yet an influence must have been diffused into the inanimate body; and not only so, but coeval, and coextensive with the existence of matter, that all the other tribes of creation, both animal and vegetable, assumed portions of it necessary for their own individual economy.

"If the pride of modern philosophy will not admit of proofs, or direct allusions from the record of inspiration, it is hoped it will stoop so low as to admit it in collateral support of a thesis partially founded on analogical facts. The pride of philosophy must be truly humbled in the view, that no greater advances have been made in the science of life since the creation of man, and of a subject constantly presented to the senses, and to the understanding also.

---

*Genesis ii. 7.

"*Illustrations.*

"It has been intimated, that this emanation of vitality is coextensive with the other properties of matter fixed on the surface of the earth, or floating in the air. It may be expedient to continue the illustration some farther. In the first place, a direct seminal influence, and sequal fecundation can scarcely account for all the phenomena of vitality noticed on the surface of the earth. Without being obliged to go into specification of much extent at this time, it may be remarked, as sufficiently proved by the investigation of geology, that various species of vegetables have been found in the transition, and after formations of the crust of the earth, which have become extinct. Again, others now vegetating are of modern origin. So of animals; natural history furnishes evidence of species, and genera of animals that have become extinct, and others have somehow sprung into existence; and surely, as far as can be discovered, without a direct seminal origin. Where are the giants of old? Here and there a petrified skeleton is found deep in the earth; and also, many unknown animals of huge dimensions, besides the mammoths, the tapirs, and hippopotami.

"We can form no conception of a seminal origin of the myriads of myriads of ephemeral insects that darken the air, or of reptiles which separate the foot of man from the surface of the earth. All springing into life at the same time, and simultaneously giving up the ghost, and becoming extinct. They appear again at uncertain intervals, and often having some slight modifications. A volume might be filled with facts on this subject; and indeed volumes have been.

"Stagnant waters imbued with some portions of albumen, gelatin, or fibrin, and probably other congenial animal substances, and when exposed to a high atmospheric temperature, soon become loaded with innumerable animacula of different genera and species, often visible, and often seen by the microscope. Neither caloric, nor oxygen, nor water combined, can produce organic beings, without a vivifying principle besides them, and then they act as auxiliaries."

"*Universal Generant Influence.*

"It is believed that those who have advocated the thesis styled spontaneous generation, have not gone so far as to designate very minutely the prolific principle, nor the substances it acts on. They commonly illustrate it by a rehearsal of

4 *

facts. It seems expedient, therefore, to follow up the subject a little further, without intending to exhaust it.

"In the first place let it be understood, that whatever scriptural allusions are made, it is not intended they should be received as strict philosophical evidence, but as collateral facts to aid the illustration of difficult subjects. We find then, that in the beginning, "the Spirit of God moved upon the face of the waters."* Let it only be noticed here, that the earth, and air both contain water. At this time, "the earth was without form." We will hardly dare to affirm, but have reason to presume, that a vital principle was at this time diffused in water, earth, and air. For we are immediately informed of the command, "and God said, let the earth bring forth grass, the herb yielding seed, and fruit tree yielding fruit after his kind, whose seed is in itself upon the earth; and it was so."† There is no mention of *seed* from which the herb germinated, but *yielding seed*, which was in the herb.

The phraseology, *whose seed is in itself*, may justly be understood as an influence imparted, and of a general kind, to the terrestrial elements, by the same Spirit which gave all the other properties to matter. The same breath which set these elements in motion, and determined the circuits of the heavenly bodies, also bestowed a principle of organic vitality, which is coeval with other properties of matter, and will be alike, or more enduring. It is as identical in its character as any other of the properties of matter, and none so fully displays the matchless beneficence of its Author. If the Pentateuch be admitted as a rule of faith in relation to divine subjects, why not in some degree respected in relation to subordinated correlative facts?

"Again, the same Creative Power, said, "Let the waters bring forth abundantly the moving creature that hath life, and fowl," &c. Also, "Let the earth bring forth the living creature after his kind, cattle, and creeping thing, "&c., and it appears that "God blessed them, saying, be fruitful, and multiply." There is no intimation of there being either in the vegetables or animals any seed from which they took root in the beginning, but the vegetables yielding seed for the nutriment of man, and the green herb for the use of animals; and both were apparently brought forth in a spontaneous manner. Although the seeds of vegetables were secondary in formation, and made for the use of animals, yet they do, for the most part, contain germinating points, like cuttings, from

---

* Genesis i. 2. † Genesis i. 11, and onward.

which other series in succession are propagated. Admitting all that can be said in favor of a seminal propagation, it is no proof against a spontaneous origin of both vegetables and animals, under particular circumstances."

To give a farther illustration of our author's physiological creed, we deem it right to quote a few clauses, containing his views of what he calls the "*suction power of the heart.*"

"6. *Cardiac Vacuum.*

"Some notices have already been made in relation to the suction power of the heart, and on capillary attraction. By way of recapitulation, we may remark, that they imply too much of mechanical principles to be in accordance with all other vital movements. The cavæ serve as reservoirs of blood in a limited manner, and suffer an undulatory motion as the right auricle contracts and dilates; the blood rushes into the auricle and ventricle as they dilate; and we freely admit that the heart is endowed with an expansive power. By this mechanism a suction force does exist, and is extended so far as the large veins extend without valves. But, it is contrary to all analogy, and all the laws of physics and of vitality, to infer, that any suction power of the heart, assisted by atmospheric pressure, can cause fluids to be absorbed by the mucous tissue of the internal viscera, or by the serous tissues. These circulations, in the capillary systems, continue for more than twenty-four hours after a cessation of the heart, and respiration. So the sap in vegetables ascends only so long as they retain any portion of vitality. The veins fill, and become largely distended when ligatures are applied to them. Furthermore, capillary circulation can be expedited in numerous ways, without any agency of the heart. The absorbents take up congenial fluids, whilst mechanical suction might take up all fluid substances indiscriminately. Finally, there is as much evidence of a vital capillary action, as there is of such an agency in the heart itself.

"Admitting, then, a limited degree of suction force by the active diastole of the heart, as influencing the circulation in the large veins without valves, and which are surrounded by an osseous encasement, we may admit another mechanical force, which has some influence on the same extent of circulation. This has reference to the process of the expansion of the thorax in the act of inspiration. The air rushes to fill the vacuum in the lungs, and a suction point is established in the whole thoracic cavity, and more space is given for the circulation generally. The jugular veins are seen to discharge their blood in the act of inspiration, and become turgid in expiration. The circulation through the lungs, also, is promoted by the dilatation of the chest, with the expanion of the vesicular tissues:—hence the panting exertions in forced respiration. Surely, in the fœtal life, the blood circulates without this force of thoracic dilatation, or any suction power, for there is a circulation before the heart is formed."

With the following descant Dr. Gallup introduces his Division of Pathology, which, to say the least of it, is sufficiently bold, uncompromising and independent. And, in these conforming, truckling, and popularity-seeking times, those qualities are meritorious and praiseworthy.

"SECTION XIII.

"1. *Preliminary Remarks.*

"Our illustrations will be conducted on the broad basis of investigation of the causes of disease, *Etiology;* the phenomena, *Semeiotice;* and the character of the morbid habit generally; or more strictly *Pathology.*

"The philosophy of medicine in all its relations, is of a peculiar character, and identical in its kind. It is in subordination to a force never found in purely physical matter; but amenable to a power which presides over organized substances, and influences their movements; alike persistive in the disturbed conditions of disease, as in the placid trains of the phenomena of health. This controlling influence is subject to various modifications from changes made in the organism by exterior circumstances, and the transition is often too easy from the normal to the disturbed state.

"The phenomena of disease are so far more intense than those of health, that the sufferer, and peradventure the medical attendant, when ignorant of the structure and functions of the organism, may be led to suppose that some additional *entity* is thrust in, and that he has new forms and sensibilities within the body, which never before existed there. The natural conclusion will be that these must be removed; and nearly the whole materia medica may be tried empirically for this purpose, and yet the suffering may still continue. This is only one of the many false views of the character of disease.

"Neither does a certain assemblage of *symptoms* constitute disease. These are only the indicators of perturbed action going on in the tissues. They speak in legible terms to those who can read, and understand the language of physical impulses on vital tissues, and organs; and the responding motions excited, and displayed by an union of apparatuses, now show a discordance, which at other times were progressing in harmony. We cannot accord with the purblind views of those who say "all physiology as far as it is hitherto known, is totally or nearly useless in explaining any thing which happens in fever." "In short, fever is a disease, the whole of the appearances of which have been in no ways accounted for."* Discouraging indeed for the long labors of the amphitheatre, and the tortures of vivisections.

"Numerous have been the false views of disease, and often so entertained by whole communities, as well as individuals. Opinion has been marshalled against opinion, and of course the treatment has been as opposite as the antipodes. Public confidence has been impaired, and even personal opinion distrusted after tedious rounds of practical experience. Medical sentiments have often been neutralized, and of course made inert; or all theorizing has been abandoned, and the mutable symptoms of disease followed up as disease itself, with a blind zeal, when they are but the mere indices of their prototypes, or the injury inflicted on the organization.

"In consequence of these jarring sentiments, a well grounded assurance in the treatment of disease has been weakened; the way opened for the entrance of impostures, and the science of medicine stigmatized as uncertain, visionary, and conjectural. Poor recompense indeed, for the labor and zeal of ages bestowed on that art, which Hippocrates esteemed divine, and which was designed to relieve the distress of man, and prolong his existence.

"Such views ought no longer to be tolerated by men of enlarged information, and who are in possession of the immense store of facts elicited by modern improvements, in all the collateral branches of medicine. These are materials for forming a new generalization of the principles of medical science; and there is the more need of this, since nothing

* Fordyce on Fever, pp. 18 and 20.

efficient has been attempted for a long time past, and the labor and responsibility is so imposing, that it has been rather evaded by many most capable of undertaking the task.

"In the outset we indignantly repel the trite, sarcastic inuendos, which have so often uncourteously been cast by many; by direct allusions to theorems advanced, having been formed from lamp-light cogitations in the closet; and the bending of facts and analogies to support a favorite hypothesis. We have been inducted by another, and more hardy routine of study. And from the full conviction of the insufficiency of every generalization hitherto attempted, have reexplored, the chartless empire of medicine; and wishing to save it from the quicksand of empiricism, have made many comparisons in the open air, in the dissecting room, and at the "bed-side of the sick." Having a few incontrovertible data, as polar stars in view, we have *endeavored* to form inductions, which will not readily be dissipated by the fire of experiment. If the arrangement is new, it is supported by facts not altogether novel.

"If we are not able on all occasions to offer a satisfactory explanation of every phenomenon of disease; yet, it is posible the outlines of a system may be designated, which if it be not perfect, still may not be false."

In his "Etiology or Pathogeny," as he himself terms it—in plain English in his views of the CAUSES of disease, our author maintains some singular notions. Of these we have a specimen sufficiently striking, in confirmation of our statement, in "Section XXV," subdivisions.

"1*st. On the general atmospheric Pestilential Afflatus.*

"REMARKS.

The history of diseases from the remotest period, even down to the present time, affords sufficient evidence of the prevalence of severe diseases at particular seasons, and far exceeding the ordinary amount. These periods continue sometimes several years, involving different countries and latitudes, or their inhabitants, more or less, in severe calamities. In some cities nearly half the population has been swept off in a short time, by one form of disease, whilst another modification, under some other name, rages in another district with nearly equal severity; the loss may be one in ten, or one in three of the inhabitants. Man knows not where to flee for safety; nor can he escape its presence; for as the poet has expressed it, "a secret venom oft corrupts the air, the water, and the land.

"*Its Extensiveness and Effects.*

"There have been seasons when the vegetable productions were blighted with mildews by some secret taint; and although luxuriant, yielded but little fruit for the sustenance of man; when likewise, the beasts of the earth, and fishes of the sea sickened and died. At the same time, or in quick succession, an invisible something seizes on the bodies of men; they also sicken and are laid prostrate, and whole communities are filled with dismay. The picture is well portrayed by our medical poet Dr. J. Armstrong, and ought to be preserved.

'Of every hope deprived,
Fatigued with vain resources, and subdued
With woes resistless, and enfeebling fear;
Passive they sunk beneath the mighty blow.
Nothing but lamentable sounds were heard,
Nor aught was seen but ghastly views of death.'

"*Ingenerate Origin.*

" The question has arisen among Philosophers, what is the medium that sustains this noxious agent, and from whence did it arise? There are some facts, and much analogy in favor of its aerial existence; and this was the sentiment of the most ancient, as well as modern pathogenist. But from whence it came, and what its entity consisted of, has been a subject of speculation and hypothesis; it has escaped every scrutiny, and every test. It received different appellations as the writer's modified views of its character dictated. Hippocrates called it *to thion*, or divine essence. Diemerbroeck styled it *pestilential germ*. Sydenham referred it to certain *occult qualities* in the atmosphere; whilst Rush and others, denominated it an *inflammatory* constitution of the air. The Arabs in allusion to the same thing as producing the cholera, call it *El Hawa*, the wind, or blast that passes. Or it may be styled a *pestilent afflatus*.

"We will chiefly contend for the fact, that certain periods, embracing from three to ten years, or more, occasionally occur, in which destructive diseases prevail, and that there exists the strongest presumption of a wide spread contaminating influence in the common air, affecting the fluids, and vital susceptibility of all organized compositions, more or less; but, from what physical influences it arises, is yet a matter of conjecture. We know not much more, or different from the allusion of the Psalmist, when he spoke of "the pestilence that walketh in darkness, and the destruction that wasteth at noon day." Dr. N. Webster has compiled a very useful epitome of medical history, in which he endeavors to establish a coincidence, and dependence of pestilent diseases on planetary influences, in connection with the agency of electricity. On a former occasion,* we were inclined to advocate the thesis; but on further reflection have grown more sceptical. From whatever source it may have an origin, it must be very extensive, as it embraces a wide range of aerial surface.

"In Section vi. 5, a suggestion was made, with a view of exciting further inquiry, that an excess of vegetative life pervading the atmosphere might, by undue stimulation, affect organized beings. On the thesis of an all prevading vital aura, Sect. vi. 6, this suggestion is raised; and on the facts, that during the pestilent periods, and in districts suffering most, there is an excess of vegetative and animal life; that myriads of insects and reptiles appear; that the intellects of men are elevated, even to a degree of impassioned ardor, and often attended with broils and wars; that the procreative powers are increased; and finally, that the entire organism shows signs of a more than ordinary state of excitation.† The effect has commonly been assigned to the cause, in the hypothesis of decaying vegetables, and putrifying animals contaminating the air; whereas it is far from probable, that the same cause, which affords an undue increase of vegetable and animal life, may stimulate the living system to excess of action, and excite disease in some form. However, there is a great weight of testimony to show, that the decomposition of vegetables or animals does not excite disease, notwithstanding so much has been advanced on the hypothesis. Sickness often follows great scarcity of vegetation and; again a luxuriance accompanies, and follows severe epidemic diseases.

*Sketches of Epidemic Diseases in the State of Vermont.

†See Tytler on the Plague.

"A considerable number of facts are put together by Professor Chapman, in an essay on the causes, &c. of epidemics; *Phil. Jour. Med. Sciences, No.* 18; which go to show very satisfactorily, that the effluvia of decomposing animal bodies do not produce disease, and nothing more than sometimes a temporary commotion. In the same work also, many facts are stated, showing an increase, and decay of numerous animals, of the lower classes, in seasons of devastating sickness. We may remark, that shallow waters have much albumen, and are filled with various kinds of vermin. He also makes this pertinent remark, in a casual manner; "It seems to me a much more rational conclusion; that this unusual fecundity is owing to the state of things of which epidemics are the effect." And further mentions the fact as occurring in yellow fever, of "the black vomit being found, during the life of the patient, so filled with animalcula as essentially to constitute that fluid." For a still more extensive collection of facts on these interesting topics, Dr. Webster's valuable work on pestilence, may be consulted with great advantage.

"We shall not urge this subject at present; but, if it be true, that there is an all prevailing vital aura, which sustains the immensity of organized beings, (Sect. iv. 6,) we may by analogy assent to the suggestion of conditions of excess, or certain concentrationslike galvanism, and the manegtic aura; and in such an event, it may no doubt spur to excess the living tissues, producing disease; and, also, cause the ocean, earth, and air to teem with life prolific, bringing into existence myriads of ephemera to flutter in the sunbeams, and stay just long enough to declare their Maker's power."

Under the same general head we have a further exemplification of these speculations, in subdivision

"6. *Remarks on the late Cholera Afflatus.*

"A brief review of the late epidemic may afford some instruction on aerial influences, as it seems to be the most extensive, persistive, and destructive of any on record. It first appeared in Asia, and extensively in the region of the Gangetic Delta, in 1817; a country where it had for ages been known to be epidemic. It appears now to have arisen with some new modifications of severity, and as if its source was inexhaustible. Our geographical allusions are intended more for atmospheric than for terrestrial boundaries. Reference may be had to various official reports from India of its extent and ravages. We can only remark that it passed in all directions, but its principal line of march was from east to west. Its devastations have reached from 20 degrees south latitude from the equator, to 65 degrees of north latitude, and more than 100 degrees of longitude, before making its transit over the Atlantic ocean.

"The particular routes of this destroyer of life have been quite irregular, but it progressed on an average about three or four miles a day westwardly. It has stopped and retrograded in its course; escaping some villages, whilst it revisited other cities, and at an unexpected time suddenly seizing on the favored ones; this is common to other epidemics. Its stay at different places was quite various, sometimes a few months, at others only a few weeks; yet hovering about, and occasionally becoming more aggravated. It loitered several years near the confines of Europe, and at length, as if it had gathered strength, in 1829 entered, visiting even the cold regions of Russia in summer and winter. In 1830 it reached England, Scotland, and made solemn progress in the south of all Europe. Before it bestrode the Atlantic, the most careful estimates of its victims amounted to fifty millions.

"The people of this western continent rested securely in the protection of the waters of the wide Atlantic. But, to us it appeared a frail barrier. In March, 1832, in an introductory lecture, the following sentiment was announced: "Even England, with her high oxygenated atmosphere, is not protected from pestiferous blasts. The cholera at this very moment proves, that science has not purged the atmosphere of malignant taints; it stalks triumphantly through every clime, and bends its proud steps towards the setting sun; decides the fate of battles, and too often defies the tardy use of medicine. Shall we claim protection from its embrace, either from our physical condition or our moral graces? Nothing short of Almighty clemency can shield us. This region has already experienced desolating sickness; a new combustion of atmospheric gases might again light up the torch of pestilence; in which event we might become the actors of other scenes and other times, as depicted by an elegant historian.

'"Dire was the tossing, deep the groans; despair
Tended the sick, busied from couch to couch,
And over all death shook his cruel dart.'"

"On the 8th and 9th of June following, (1832,) the Asiatic Cholera, as it had been called, showed its frightful aspect at Quebec; fifteen sickened, and seven died on the ninth; some of these were emigrants just arrived, and some were citizens. The belief of its communication from one person to another, by those who knew not its character, excited great alarm, as it had done wherever it had appeared. Its rapid spread to Montreal, New York, Detroit, the great West, and South, and even South America, cannot be detailed here, but may be learned from many excellent treatises written by American physicians. Finally, the cholera afflatus in a period of about seventeen years, had nearly embraced every section of the earth, destroying people of different temperaments and habits. In 1835 it still loitered in many places, but was not so severe.

"One fact in connexion with the first appearance of the cholera on the American shore ought not to be omitted; which is, that there was an easterly or north-easterly wind, which prevailed very constantly for four weeks previous to the 11th of June, 1832, when it ceased. There was an almost continued dripping, and it might be called a rainy season, together with a degree of coldness which threatened to blight the prospects of agriculture. Surely the coincidence of so long continued wind, and the immediate appearance of the cholera, are worthy of notice.

"There appear to be numerous circumstances which favored the progress of the disease in particular places, and amongst particular classes of people, which cannot be noticed now. Notwithstanding it followed the low courses of fresh water, and seized on the profligate and enervated, yet afterwards it did visit high and dry regions, and affect the most healthy and circumspect part of society. The local and exciting circumstances will be considered in their places; but none of these can produce the disease without the predisponent pestilent afflatus."

Dr. Gallup is unfriendly to the belief, that masses of animal and vegetable matter, in a putrid condition, engender disease. In opposition to that doctrine he advances what he denomi nates "*Instances in proof*," which are as follows.

"In support of this last proposition, we will briefly advert to a few out of many facts which might be presented.

"In all parts of our own country, as well as in others, there are numerous slaughter-houses; and at every such place there arises a stench very offensive to those not accustomed to it; and yet, the people engaged in the work, or visitors, are not made sick by it; and it is even confidently asserted that those people are not so likely to be affected as others when sickness prevails.*

"In the anatomical dissecting rooms, now numerous even in this country, and greatly multiplied in Europe, no disease has ever been assigned as originating from them, although it had ever been supposed, that decompositions of human organization afforded the most noxious effluvia.

"In every farmer's barn-yard, in the country, in every house-yard, also, in villages and cities, there is usually going on decomposition in the warm seasons of the year, and often the effluvia are very offensive, and yet no disease prevails, perhaps, in six years out of seven; but, if there should be disease, how familiar it is to refer it to those disgusting gases? These offensive gaseous productions are chiefly hydrogen, sulphureted and carbureted hydrogen, ammonia, and carbonic acid gas: none of which, separate or combined, ever produce a pyrectic habit, even where greatly evolved in chemical processes.

"During the impulse of national fervor that afflicted revolutionary France, the existing powers authorized the breaking up of the graves of the ancient sovereigns at St. Dennis, as a kind of revengeful insult. It is represented by Mr. Howard, that there arose from the tombs a thick, dark vapor, extremely offensive, which produced sudden depression, and even fatal asphyxia in some of the wretches engaged in it, but no defined pyrectic disease ensued. All these kind of gases impress the nervous system of external relation, without very essentially affecting the great system of nutrition, the seat disease. (Sect. xvi. 3.)

"Another instance has often been alluded to by etiologists, as occurring at Paris, and published by M. Thoset, in the Jour. de Physique, 1791.† It consisted in the removal of

* Dr. W. F. Edwards, "On the influence of physical agents on Life, p. 186, Phil. Edit. states, that at Montfaucon, in one of the greatest slaughtering establishments of horses, dogs, cats, &c. in the environs of Paris, "that the cholera affected scarcely any of the workmen." Also, by the advice of Dr. Duchatelet, that the workmen and their families, "are in general remarkably healthy."

† Phil. Jour. of Med. and Science, No. 17, p. 376.

the cemetery of St. Innocens, which covered about two acres of ground in the centre of the city, estimated to contain six hundred thousand bodies, which had been accumulating for six centuries; it occupied two years in removing it. The stench was quite insupportable, and often the mephitic vapors affected some of the laborers with dizziness, syncope, tremors, and even asphyxia, yet no fever occurred, nor any endemic prevailed.

"Other instances of the removal of cemeteries might be mentioned, and with similar results; and also, we might take into the account the manufactories of animal substances as the making of adipocire, candles, soap, &c. The extensive fisheries also; foul wharves, giving out complicated and disgusting effluvia, might he adverted to, and every reference would only confirm the fact, that neither endemic nor epidemic disease, has ever been shown to be generated by them, and that these gases exist in full profusion during the most healthy periods. Professor Chapman has done a service to medicine, in collecting many facts to support the thesis in his Thoughts on the Causes, Phenomena, and Laws of Epidemics, &c.

"Having a presentiment that the enlightened community of medicine will readily assent to the foregoing positions, this part of our subject may be relinquished, whilst we enter upon the other, and by far more laborious part."

It has long been a question very strenuously contested, whether *cold* be in its nature, a *sedative* or a *stimulaut?* And were the contest decided by the *number* of polls that have taken sides on the subject, the *sedative* doctrine would abundantly triumph. But we are not convinced that the *preponderance* of sound philosophical testimony, in favour of the *stimulant* doctrine, would not be equally decided and abundant. And if we mistake not the signs of the times, on the subject, the latter doctrine is now on the ascendant. Be this however as it may, it appears from the following clause, in his "Etiology," that our author is a believer in the *stimulancy* of cold.

"*Cold considered Pathologically.*

"One of the most unfortunate of all errors that has ever crept into pathology, is the hypothesis, that cold acts as a sedative on living animal tissues; or, that it debilitates them. It will on the contrary be contended, that cold is a positive agent, and acts on vital tissues as an astringent, or tonic. It is then quite easy to conceive, that an inability of function may be the immediate effect of its application, in any considerable degree. Yet, when high heat has been applied, the admission of cold becomes indirectly invigorating. Cold in an incompatible degree impedes the nervous energy, also the circulations and functions of organs generally. The condensation of tissues affects the minute nervous fibrillæ producing enervation, and insensibility to ordinary stimulations; it produces an inability of nervous energy, whilst the simple tissues are condensed.

"Cold applied to the surface, simultaneously affects the internal tissues. A state of enervation is more or less every where produced, yet often unequal. Innervation and circulation are impaired. Some tissues for a time may act in excess; but, when the effects of cold are in any considerable degree, the deficiency of function may be so great, that the instinctive energies cannot prevail, and life may be extinguished, even whilst the inmost tissues are held in rigid endurance."

On our author's "Synopsis of Nosography" we can dwell long enough only to say, that, for its length (and it is not short) it is one of the most elaborate and laborious American productions, in medicine, that our country has witnessed. It possesses nearly all the learning, but not all the unapplicableness to practical and useful purposes, which characterizes Dr. Good's monument of erudition and pedantry, patience and perseverance, on the same subject. Without any further notice of it however, we must pass to our author's third "Division" of his "Outlines;" his

THERAPEUTICS.

Here again our remarks must be few and brief, this article being already protracted beyond its intended limits. Although, under a critical examination, we could find much to except to, in our author's "Therapeutics," it is perhaps notwithstanding the least exceptionable, and the most useful "Division" of his work. True; without great modification, the practice he recommends would be altogether inadmissible in the treatment of our western diseases. The reason is plain. It is suited to the complaints of a far different situation and climate—we mean New England. In speaking respectfully of it therefore, we regard it only in its relation and aptitude to the maladies of that region. As easily could Dr. Gallup devise and mature a successful scheme of buffalo and bear-hunting, without ever having seen a buffalo or a bear, a wilderness or a prairie, as such a scheme for the treatment of the fierce diseases of the Mississippi Valley, without having practised on them. In the treatment of the complaints, with which he has been conversant, we are inclined to believe him a skilful practitioner. Under the head of "*Pharyngitis, Laryngitis, and Tracheitis*" he has given a few paragraphs, which will be read, as we feel persuaded, with a deep interest, by every American. We allude to his exposition of the errors, neglect, and inefficient practice, which led to a fatal termination in the last illness of Washington. The following are his remarks on that subject.

"The medical reputation of the United States suffered not a little obscuration, and the nation an irreparable privation, in the death of General Washington at the very close of the last century, by an imbecile, doubting management. It is not pretended but that he was himself in fault; so like many others in high stations, skilled in every thing, except what relates to the character of disease. After an exposure in December, on horse-back, he had hoarseness and sore throat, but called it a *cold*, and said in the evening, "Let it go as it came," as stated in Paulding's life. So nothing was done. He read newspapers on the evening of Friday, and "had been remarkably cheerful all the evening." Went to bed as usual. Between two and three o'clock had a rigor; but made no alarm, and still had nothing done. Early in the morning of Saturday was found, "breathing with difficulty, and hardly able to utter a word." Whenever he attempted to swallow, he became "convulsed and almost suffocated;" at the same time "some phlegm followed" the return of vinegar, molasses, &c. attempted to be swallowed. This was, no doubt, a complicated case of laryngitis, associating other contiguous tissues, and producing pharyngitis, with some appearance of tracheitis.

"An overseer attempted to bleed, without any previous preparation by external warmth; he obtained half a pint, when Mrs. Washington interfered, and had it stopped. Dr. Craig arrived next day, at nine o'clock, A. M. He put an epispastic on the throat; took more blood; again bled; and repeated at three o'clock, P. M. No account is given how much, but it "ran very slowly, appeared very thick," and no doubt it might have been added, was very dark. Bleeding without other adjuvants is commonly useless. However, "calomel and tartar emetic were administered, without effect." At five o'clock, P. M., he said, "I find I am going, my breath cannot contiue long." He languished until about ten o'clock, P. M., and expired December 14, 1799. From the time of the rigor about twenty hours.

"No doubt this case demanded the most efficient constitutional as well as local treatment, and an at early period. At a later period there might have been some chance of success. It might be inquired, at what later period of the disease there existed a fair chance of relief. His case required diligent attention at the time he went to bed; then it is probable a pediluvium, and an emetic followed by sweating, would have been sufficient; especially with venesection after the surface became warmed.

"At the time of the rigor, the morbid changes had proceeded so far as to require very vigorous measures. He then ought to have taken the universal warm bath for twenty minutes; or mild steam well applied might have been a substitute, but never an equivalent. Then copious bleeding, even to faintness, closely followed by an emetic of tartarized antimony. Warmth continued with diluent aromatic drinks, as hyssop, pennyroyal, &c., followed by copious and continued sweats. Instead of an epispastic to the throat, as was done in this case, an extensive emollient cataplasm of linseed meal and wheat bran; first anointing the throat with the anodyne saponaceous balsam, or some camphorated oil. Also, cataplasms with mustard seed flour, to the whole of the feet. All should have been done and repeated in the most perfect manner, under the immediate inspection of the physician.

"But, no physician saw him, to prescribe, until Dr. Craig arrived at nine o'clock, A. M. Important time had been lost; the appearances were inauspicious. Still, however, the patient ought even at this late hour, and in a disease making awful strides, to have had the chances of relief, and this was the last hour that presented much prospect. As nothing can be gained in these cases if caloric in a humid form to the surface is neglected, he ought to have had all the measures diligently applied, as mentioned before, at three o'clock, the time of the rigor.

"An emetic was indispensible at either of the periods. Although the difficulty of swallowing was great, yet at either period it is probable a little of a concentrated solution of tartarized antimony might have been got down, or at any rate by frequent trials, some might have been absorbed in the fauces. At the same time the epigastrium might have been bathed every ten minutes with a warm and strong solution of the same. Furthermore, a scruple of the same article in solution, might have been injected as an enema. By persevering, there is but little doubt, emesis might have been effected. The influence of this process on the general capillary systems was as much needed, as its utility in dislodging phlegm from the trachea."

We regret that a want of room prohibits us from giving extracts from the "Outlines" explanatory in full of our author's mode of treating pulmonary consumption. The treatment is bold, and, according to his report of it, unusually successful. In consequence of this, he speaks of the complaint as being much more tractable, than it is generally considered—especially if it be encounted at an early period. The remedies on which he chiefly depends, are the warm bath and blood-letting *often repeated*, and sometimes long persevered in, emetics regulated exercise, and a diet mild, and easy of digestion.

The Doctor illustrates his practice and its success in this complaint, by a detailed report of his treatment of two cases, highly threatening, but which terminated favorably. His entire account of the malady we deem curious and interesting, and earnestly recommend it to the attention of physicians.

But we must here close our article. And we do so by again repeating, that Dr. Gallup's "Outlines" contain much matter that is valuable, mingled with not a little that is totally useless—if not worse. By physicians sufficiently enlightened in their profession, by experience and observation, to be able to adopt and reject, in the contents of books with judgment and skill, the work may be read with no little benefit. And the same may be done by medical pupils, whose studies are superintended by vigilant, faithful, and competent preceptors. But, without such superintendence, we cannot recommend the work as a text-book for the elementary studies of youth.

In conclusion: there are two points (the *beginning* and the *end* of his paper) where a reviewer may speak not only of the general character of the *work* which he reviews, but also of the nature and character of the *subject* of which it treats. And as we did not touch on the latter topic, in the *commencement* of this article, we shall briefly advert to it in its close.

The "Institutes of Medicine" embracing, as they do, the principles and philosophy of *all the other branches of the profession*, are, of course, the *highest and most important* of all. Without them medicine would not be an *embodied science*; but only an assemblage of kindred, but disjointed, and empirical pursuits. The Institutes are the life-blood—the soul of the profession, without which it would be even worse than inanimate and inefficient—it would be a scheme of *haphazard* action, much more likely to do mischief than good. It would resemble navigation, deprived of the aid of its chart and compass. Its making good its course to the wished-for haven, would be a matter of accident—the chances against its arrival being *multitudes to one.*

If this representation be true, (and who will gainsay it?) to write a work on the Institutes of Medicine is an *arduous*—it would be hardly extravagant to say—a *mighty* undertaking. Its accomplishment in a style and degree of *general* excellence worthy of its subject requires talents and attainments, industry, perseverance, and a lofty ambition, which belong to but few of the medical profession—or of *any* profession.

The production of a competent work on the Institutes requires in its author a thorough, minute, and accurate knowledge of every other branch of the profession. Nor is this all. Not only must he be in possession of the almost boundless multitude of *requisite facts.* He must have so clear and correct a perception of their *relations to each other, and to their aggregate*, as to be able to deduce from them the sound abstract, and general principles, which they are calculated to sustain. To be qualified to do this, he must be amply endowed with *all* the intellectual faculties, especially with those of the highest order; (we mean the *reflecting*) and those faculties must be strengthened and rendered dextrous in action, by assiduous and skilful cultivation and practice. In a word, he must be, in the full and genuine meaning of the term, a MEDICAL PHILOSOPHER. And of such characters the number that has adorned the profession of medicine is small. It would not be extravagant to say, that, since the days of Hippocrates, there has not been one for each succeeding century—not one we mean of the *highest rank*, and competent to the achievement we are now considering. So true is this, that there does not exist at present, in any language with which we are acquainted, a single work on the Institutes of Medicine, worthy in all respects of the momentous subject. Many such works are high in merit; but they are all below the standard they should attain. Nor do we mean by this assertion to disparage the Medical Faculty of either the present or former times. Our only object is, to express our own estimate of the exaltedness of MEDICAL PHILOSOPHY, and the arduousness of ascent to the pinnacle where it perches. C. C.

